# Cholesterol and the Safety Factor for Neuromuscular Transmission

**DOI:** 10.3390/ijms20051046

**Published:** 2019-02-28

**Authors:** Igor I. Krivoi, Alexey M. Petrov

**Affiliations:** 1 Department of General Physiology, St. Petersburg State University, St. Petersburg 199034, Russia; iikrivoi@gmail.com; 2 Institute of Neuroscience, Kazan State Medical University, Butlerova st. 49, Kazan 420012, Russia; 3 Laboratory of Biophysics of Synaptic Processes, Kazan Institute of Biochemistry and Biophysics, Federal Research Center “Kazan Scientific Center of RAS”, P. O. Box 30, Lobachevsky Str., 2/31, Kazan 420111, Russia

**Keywords:** skeletal muscle, neuromuscular transmission, safety factor, cholesterol and lipid rafts, oxysterols, synaptic vesicle cycle, quantal release, Na,K-ATPase, nicotinic acetylcholine receptor

## Abstract

A present review is devoted to the analysis of literature data and results of own research. Skeletal muscle neuromuscular junction is specialized to trigger the striated muscle fiber contraction in response to motor neuron activity. The safety factor at the neuromuscular junction strongly depends on a variety of pre- and postsynaptic factors. The review focuses on the crucial role of membrane cholesterol to maintain a high efficiency of neuromuscular transmission. Cholesterol metabolism in the neuromuscular junction, its role in the synaptic vesicle cycle and neurotransmitter release, endplate electrogenesis, as well as contribution of cholesterol to the synaptogenesis, synaptic integrity, and motor disorders are discussed.

## 1. Introduction

Reliable transmission of brain commands through motor nerve to skeletal muscle and physical activity are required to maintain both motor function and muscle mass and, thus, important to ensure a healthy life. Skeletal muscle neuromuscular junction (NMJ) represents specialized region where the axon of a motor neuron establishes synaptic contact with a striated muscle fiber. The efficiency of the neuromuscular transmission strongly depends on a variety of pre- and postsynaptic factors [1,2].

Proteins that regulate quantal neurotransmitter release are clustered at specialized regions of the presynaptic membrane, called active zones (AZs). AZs represent highly specific compartments that contain synaptic vesicles (SVs) and serve as a molecular platform for precise spatial and temporal control of vesicle fusion and quantal neurotransmitter release [3]. Following release from the motor nerve terminals, neurotransmitter acetylcholine (ACh) diffuses across the synaptic cleft; during this travel ACh molecules are partially hydrolyzed by the enzyme acetylcholinesterase (AChE). The remaining ACh molecules interact with the postsynaptic nicotinic acetylcholine receptors (nAChRs), which are distributed at very high densities (~10,000/μm^2^) at the crests of the primary postsynaptic folds of the adult NMJ [4]. Molecules of ACh released from one SV (a ‘quantum’ of transmitter) activate approximately two thousands of nAChRs, leading to opening cationic channels of the nAChRs and a net influx of positive ions. The latter produces a local transient (within milliseconds) small (~1 mV) depolarization, called the miniature endplate potential (MEPP). MEPPs occur spontaneously without presynaptic action potential (AP); the frequency of MEPPs reflects spontaneous neurotransmitter release. Motor neuron activity is translated to firing of APs, which, upon arriving to nerve terminals, trigger release of the multiple SVs. As a result, greater levels of neurotransmitter in synaptic cleft produces more depolarization than MEPP called the endplate potential (EPP). This local depolarization extends towards the depths of the secondary synaptic folds where, in turn, the voltage-gated sodium channels are located. The sodium channel’s opening triggers the AP generation, thereby transforming the local EPP into propagating AP of the muscle fiber.

The fidelity of the neuromuscular transmission will depend on whether the EPP amplitude exceeds the threshold for muscle AP generation. The EPP amplitude depends on the amount of ACh quanta released (quantal content) and individual quanta size, AChE activity, the density of the nAChRs packaging, and the resting membrane potential (RMP). The AP generation threshold (membrane excitability) also depends on the RMP [5]. The term ‘safety factor’ refers to the ability of neuromuscular transmission to be effective and is quantified by the ratio of the EPP amplitude to AP generation threshold [1,2]. In different muscles, depending on their specialization, the value of the safety factor ranges from 2 to 5, however, it can decrease under various physiological and pathophysiological conditions such as fatigue, injury or diseases [1,6,7].

A normal segregation of plasma membrane lipid phases is vital to the maintenance of membrane fluidity, curvature, ion channel, transporter functions, as well as compartmentalization. Importantly, membrane proteins that encompass about 50% of the cell membrane are often cocrystalized with membrane cholesterol [8]. Cholesterol’s presence in membranes regulates the function of many proteins, including transporters, receptors, and ion channels. There are two main ways of how cholesterol can affect protein properties. First mechanism is through specific binding of cholesterol with high affinity sites. This may contribute to control of protein conformation and function. Second mechanism is dependent on the nonspecific physicochemical influence of cholesterol on membrane fluidity and thicknesses that consequently affects numerous protein-dependent processes. Distinguishing between mechanisms of cholesterol action can be challenging [8,9].

The basic principles of lipid-protein interactions have still not been completely elucidated. Cholesterol-associated changes impact membrane fluidity and stiffness, lateral lipid diffusion, protein mobility, excitability, and a variety of other key functional membrane properties including fusion and fission. In addition, membrane cholesterol modulates interactions between the lipid bilayer and ions environment as well as the interplay between plasma membrane and underlying cytoskeleton suggesting the involvement in mechanotransduction. Cholesterol is also a key molecule in the formation of lipid rafts known as molecular platform involved in numerous cellular processes like apoptosis, signaling, and cell differentiation. Little is known, however, about the precise consequences of changes in membrane cholesterol levels of different cell type. Notably, responses to membrane cholesterol enrichment or depletion vary depending on cell type and can be very complex and unpredictable [10,11,12,13,14].

While the essential role of cholesterol for many vital functions is well documented, much less is known about its role at the NMJ, in particular, in maintaining the safety factor for neuromuscular transmission. The review focuses on the crucial role of cholesterol to maintain a reliability of neuromuscular transmission in health and diseases.

## 2. Cholesterol Production

Cholesterol production is energetically expensive and requires more than 30 enzymatic reactions. Different cells in nerve system have a different capacity to produce cholesterol. Oligodendrocytes are characterized by a higher capacity than astrocytes which in turn, had at least 2–3-times higher ability to cholesterol synthesis than neurons [15]. During nerve system development the intensity of cholesterol synthesis by different cells undergoes significant changes. Cholesterol production by oligodendrocytes is responsible for the great increase in cholesterol content at period of active myelinization with its peak during the first postnatal weeks. After myelinization, synthesis occurs at low rate in nerve system of adult mammalians [16]. Before glial cell differentiation, a neuron could cover its own cholesterol requirements by producing cholesterol. In the postnatal period, neuronal ability to cholesterol production is strongly reduced and neurons rely on the cholesterol mainly synthesized in glia [17,18].

Neuronal requirements for cholesterol are extremely high, as it is indispensable for formation of synaptic membranes [17]. Studies in central and neuromuscular synapses suggest that all steps of SV cycle (exocytosis, endocytosis and vesicular traffic) could be dependent on cholesterol content in presynaptic membrane and membrane of SVs [19,20]. The presynaptic part of NMJs is far from the motor neuron soma, where the main biosynthetic apparatus is located, and this part is adapted to translate high frequency patterns of motor neuron activity to muscle. To prevent the loss of connectivity, pre- and postsynaptic compartment are tightly stickled by extracellular matrix and presynaptic part is covered by Schwann cell. Accordingly, mechanism of cholesterol delivery to neurons and maintenance of cholesterol steady state level in synapse should be crucial for neurotransmission, especially in presynaptic compartment of NMJs. The best candidate to provide NMJs with cholesterol is terminal Schwann cell, which can produce apolipoprotein (particularly, apolipoprotein E) and cholesterol containing particles. Consistent with this, stimulation of apolipoprotein receptors are important for the formation, maintenance and regeneration of peripheral NMJs [21,22].

Skeletal muscles comprise approximately 50% of total body weight and contain T-tubule membranes, enriched with cholesterol. Cholesterol sources for muscle are both local biosynthesis and uptake from the circulation. The later has a predominant relevance and high-rich diet could lead to enrichment of muscle fiber membranes with cholesterol [23,24]. However, certain basal level of local cholesterol production in the skeletal muscle is probably essential as inhibition of cholesterol synthesis (with statins) caused muscle fatigue and weakness. This may be linked with both the high requirements of cholesterol for muscle membrane remodeling, and the importance of cholesterol biosynthesis intermediates (e.g., ubiquinone, dolichol, farnesyl, and geranyl pyrophosphates) [25]. Additionally, skeletal muscle can store cholesterol in the form of cholesterol esters in lipid droplets, and their accumulation could be accompanied by myopathy [26]. Cholesterol elimination from muscle membranes theoretically may be linked with muscle activity. Membrane cholesterol is susceptible to oxidation by reactive oxygen species (ROS) [27] and ROS produced during muscle loading could oxidize membrane cholesterol, leading to cholesterol elimination in the form of oxysterols. Oxysterols can activate nuclear liver X receptor in skeletal muscle [28,29] and, thus, trigger increased cholesterol synthesis and (or) uptake to compensate cholesterol elimination. Therefore, skeletal muscle cholesterol metabolism probably relies on balance between cholesterol synthesis, consumption, storage, and elimination.

## 3. Cholesterol and Quantal Neurotransmission Release

Motor nerve endings are specialized on fast neurotransmitter release in response to arriving AP. Neurotransmitter molecules are packed into SVs which distributed into cytoplasm near sites of exocytosis. Small population of SVs is docked at the presynaptic membrane in specialized regions, AZs. Some of these SVs, consisting of ready releasable pool, are able to fuse with short delay after Ca^2+^ influx through voltage-gated Ca^2+^ channels located within membrane of the AZ. After exocytosis, lipid and protein components of vesicular membrane are internalized by endocytotic mechanism. Newly formed endocytotic vesicles may be repeatedly used for neurotransmission after refilling with neurotransmitter. Thus, synaptic activity is accompanied by SV exo-endocytotic cycles (recycling) [19,30]. These cycles put several challenges for presynaptic machinery. First, fast and intensive exocytosis and compensatory endocytosis require changes in membrane curvature and rigidity; second, SV and AZ membranes have specific protein–lipid composition, which should be maintained, despite fusion of the SVs into the presynaptic membrane; third, AZ, and surrounding presynaptic membranes (peri-AZ) should have different properties to allow fast exocytosis and relatively fast endocytosis; forth, during innervation and reinnervation membrane properties should be different to easily adapt shape of distal axon to target postsynaptic compartment. Of course, protein-based mechanisms are primary responsible for resolving these challenges, but cholesterol as one of the main component of presynaptic vesicle membranes are involved in maintenance of presynaptic function [31].

Initially, in electron microscopic study it was found that sterol-binding antibiotic filipin did not form complex with cholesterol in AZs in the frog nerve terminals. At the same time, filipin–sterol complexes were detected in most axonal areas, including regions of nerve terminals lacking AZ, and membranes of Schwann cell membranes [32]. Moreover, during degeneration or regeneration, despite AZ disorganization or presence in primitive form (without normal double row organization) filipin–sterol complexes were excluded from these membrane sites, while Schwann cells occupied synaptic gutters displayed high abundance with filipin–sterol complexes [33]. At the same time, more intensive filipin staining was detected in axons of crayfish, frog, mouse, and rat, suggesting cholesterol abundance in nerve terminals [34,35,36]. This cholesterol could organize membrane microdomains, lipid rafts. Indeed, axonal synaptic membranes of frog, mouse and rat showed a high intensity of staining with lipid raft markers [35,37,38,39]. Moreover, acute cholesterol depletion with methyl-β-cyclodextrin (MβCD) led to decrease a lipid raft labeling [37,39]. All together, these results suggest that the membrane regions of AZ can have a relatively low cholesterol content, but other regions of the presynaptic membranes are cholesterol rich and contain lipid rafts, which could organize fence around AZ. Thus, presynaptic membrane cholesterol could have functional rather than structural meaning for SVs exocytosis.

It is conceivable that cholesterol within and in close vicinity of exocytotic sites could have a regulatory role and be kept lower for maintenance of regulation range. In a pioneer study by Zamir and Charlton [34] it was found that cholesterol depletion blocked evoked neurotransmitter release, without marked postsynaptic effects. Suppression of evoked neurotransmitter release was linked with hyperpolarization of presynaptic axon and lack of AP propagation, whereas Ca^2+^-dependent exocytosis functioned and spontaneous exocytosis was even enhanced. All these effects were reversed by cholesterol supplementation. This study points to importance of cholesterol level for presynaptic transmitter release. Cholesterol depletion suppressed evoked neurotransmitter release, but increased spontaneous exocytosis in frog and mice NMJs as well as central synapses [40,41,42,43,44,45]. Thus, cholesterol level could determine the ratio between spontaneous and evoked neurotransmission. In context of NMJs, it means that lower cholesterol content could lead to decrease safety factor due to suppressed AP-evoked exocytosis and increased energy expenditure and desensitization of postsynaptic receptors as a result of upregulated spontaneous neurotransmitter release. Notably, that enhanced spontaneous exocytosis could facilitate uptake of extracellular macromolecules, as was shown for botulin neurotoxin A [46]. Speculatively, it may be a part of homeostatic response to cholesterol depletion, because compensatory endocytosis following by exocytosis could internalize of cholesterol-containing lipoprotein particles, which are likely main source of cholesterol for motor nerve terminals.

Several signaling mechanisms could be responsible for increased spontaneous exocytosis after cholesterol depletion. In frog NMJs, decreased cholesterol content led to increase in NADPH oxidase-dependent ROS production, which stimulated an increase in cytosolic Ca^2+^ (probably via TRPV channels) and, subsequently, calcineurin (phosphatase PP2B) activation, which, in turn, facilitates the involvement of SVs to spontaneous exocytosis. Under these conditions, increased ROS production led to marked lipid peroxidation of synaptic membranes [41]. Moreover, activation of protein kinase C in response to cholesterol depletion can support SV exocytosis in full mode, whereas inhibition of protein kinase C (but not phospholipase C) converts exocytosis to kiss-and-run mode (when transmitter release occurs through transient fusion pore) in frog NMJs [42]. Involvement of protein kinases A and C in the effect of cholesterol depletion on spontaneous neurotransmitter release was also observed in central synapses [47]. Notably, cholesterol depletion may provoke spontaneous exocytosis from separate SV pool, which reluctantly participates in evoked neurotransmitter release in frog NMJs [44]. Thus, presynaptic cholesterol level acting via signaling mechanism can limit both spontaneous neurotransmitter release and overactivation of protein kinases, phosphatase PP2B as well as production of ROS and increase in cytosolic Ca^2+^. This housekeeping role of membrane cholesterol in NMJs could maintain neuromuscular transmission and signaling at steady state level.

Molecular study indicates that SV membrane contains very high amount of cholesterol [48]. Decreased cholesterol content in membranes of SVs in Drosophila, frog and rat NMJs as well as central synapses led to inhibition of SV endocytosis and recycling [36,40,49,50]. Furthermore, in Drosophila NMJs, cholesterol extraction from SV membranes caused dispersion of SV proteins (synaptotagmin, VGLUT, and CSP) throughout presynaptic membrane and disturbance of actin polymerization, suggesting requirement of vesicular cholesterol for maintenance of protein composition of SVs and regulation of cytoskeleton dynamic [51]. Therefore, vesicular cholesterol is essential for SV endocytosis and could serve as glue for keeping SV proteins and lipids in one membrane microdomains (lipid rafts), which are selectively trapped by endocytotic mechanism [35,52]. Along with this, antiganglioside GM1 antibodies (hallmark of Guillain–Barré syndrome leading to acute motor axonal neuropathy), which recognize components of lipid rafts, are intensively uptaken by motor nerve terminals, counteracting damage of the nerve terminals [53].

SV exocytosis is a major mechanism of neurotransmitter secretion. However, nonvesicular (nonquantal) secretion of neurotransmitter also occurs in NMJ and central synapses. The functional role of this type of ACh secretion is elusive. In rat NMJs, depletion of cholesterol from SV membranes led to an increase in nonquantal ACh release due to the incorporation of neurotransmitter transporters (in particular, vesicular ACh transporter) into the presynaptic membrane and enhancement of ACh/H^+^ exchange [36]. Thus, cholesterol level in SVs could limit neurotransmitter leakage via nonquantal release.

## 4. Effects of Cholesterol Derivatives on Neurotransmitter Release and Synaptic Vesicle Cycle

Cholesterol is oxidized by ROS and specific enzymes to oxysterols, which could modulate multiple processes, linked with inflammatory reactions and neurodegeneration [19,54,55]. Synaptic transmission in NMJs of frog and mouse was a high sensitive to different oxysterols and enzymatic cholesterol oxidation [37,38,39,56,57]. 24S-hydroxycholesterol (24HC) is a product of cholesterol metabolism, which is generated specifically in brain and passes across a blood–brain barrier into the circulation. The levels of 24HC in the circulation are significantly changed at early and (or) advanced stage of neurodegenerative disease [15,19,54]. 24HC (0.4–4 μM; 1/3 h-application) enhanced synaptic transmission in mice NMJs due to increase in a rate of SVs recycling. The mechanism of 24HC action was linked with decreased NO production, likely by endothelial NO synthase [57]. Activity of this enzyme is dependent on its association with lipid rafts, partially, via caveolin-dependent mechanism.

In an amyotrophic lateral sclerosis (ALS) mouse model carrying a mutant superoxide dismutase 1 (SOD1^G93A^) 24HC also decreased NO production, but led to an opposite effect on neuromuscular transmission, because the role of NO is likely reversed in ALS mice as compared to wild type mice. Increased NO synthesis promoted SV exocytosis during intense activity in SOD1^G93A^ mice, but it suppressed the exocytosis in wild type mice [39]. Probably, attenuation of NO production was mediated by increased lipid raft integrity in response to 24HC. Indeed, disturbance of lipid rafts prevented the effect of 24HC on NO synthesis [39]. However, it is unclear why NO had opposite actions in the ALS versus wild type mice. It is conceivable that increased mitochondrial ROS production [58] could invert the effect of NO at the NMJs of SOD1^G93A^ mice. For example, peroxynitrite formed from the reaction between NO and ROS can promote exocytosis probably due to direct modification of AZ proteins SNAP-25 and Munc18 [59]; while NO, acting as retrograde messenger, may inhibit neurotransmitter release due to activation of guanylyl cyclase/cGMP pathway [60] or direct S-nitrosylation of SV fusion-clamp protein complexin [61].

Another oxysterol, 5α-cholestan-3-one (5Ch3), is produced as an intermediate sterol in the biosynthetic pathway for cholestanol. The levels of 5Ch3 were increased in *Cerebrotendinous xanthomatosis* patients [62], having muscle dysfunction. 5Ch3 (0.2 μM) decreased the amount of SVs which were actively involved in neurotransmitter release in mouse NMJs. The effect of 5Ch3 was linked with decrease in lipid raft integrity and was dependent on membrane cholesterol content [37]. Similarly, 5Ch3 reduced lipid raft integrity and the number of SVs participating in exo- and endocytosis during synaptic transmission in frog NMJs [38]. In contrast, structurally similar oxysterol olesoxime (cholest-4-en-3-one, oxime; TRO19622) increased evoked ACh release as well as the number of SVs involved in exo-endocytosis and the rate of SV recycling. Moreover, olesoxime was able to increase lipid raft integrity in frog NMJs [38]. Note that olesoxime is potential neuroprotective compound in models of ALS, multiple sclerosis, Parkinson’s, and Huntington’s disease [63,64,65,66]. These data show that these oxysterols induce marked different changes in neuromuscular transmission which are related with the alteration in SV cycle and lipid raft behavior.

Similarly, oxidation of endogenous cholesterol by cholesterol oxidase significantly impaired lipid raft integrity as well as affected mode of SV exocytosis (toward to kiss-and-run mechanism) and disturbed SV clusterization [56]. The effects of cholesterol oxidase on SV cycle were different from cholesterol depletion [40], suggesting that oxidative cholesterol derivative (cholest-4-en-3-one) could mediate action of cholesterol oxidase. Taken together, oxidized cholesterol metabolites could present a new class of presynaptic neurotransmitter release modulators, which may contribute to adaptation of muscle activity to current physiological status of organism.

## 5. Cholesterol and Proteins Involved in Synaptic Vesicle Cycle

Cholesterol-interacting proteins could serve as transducer of changes in local cholesterol level to presynaptic processes. Cholesterol microdomain can clusterize Ca^2+^ channels (e.g., N-, L-, and P/Q types) in the presynaptic membrane of neuronal cells, affecting distance from the channels to the site of exocytosis and, thus, neurotransmitter release [67,68,69]. Also, a main Ca^2+^ sensor—synaptotagmin 1—triggers SV exocytosis and is a lipid raft resident [70]. Studies with cholesterol depletion suggest that neurotransmitter transporter distribution and (or) their activity in presynaptic terminals could be dependent on cholesterol availability [36,71,72,73]. Also a vesicular H^+^ pump, which creates a proton gradient for neurotransmitter flux into SV, was also found in cholesterol microdomains and cholesterol depletion attenuated the H^+^-ATPase activity [74]. Cholesterol depletion could also suppress SV swelling mediated by coordinated activity of H^+^ pump and water channel aquaporin-6 [75]. Several studies suggested that clusterization of syntaxin, an essential component of exocytotic machinery, is affected by membrane cholesterol [76] and depolarization of synaptosomal membrane increases redistribution of syntaxin into lipid raft fraction [77]. Furthermore, cholesterol may be a part of the fusion pore, connecting lumen of SV with extracellular space, and increasing cholesterol content favors fusion pore opening [78,79]. This is in agreement with extremely high cholesterol content (40 mol%) in SVs [48]. Interaction of most abundant SV protein, synaptophysin, with cholesterol could be important for SV endocytosis [52]. Interestingly, a mutation in DJ-1 (a genetic factor for early-onset autosomal recessive Parkinson’s disease) impaired SV endocytosis, without inducing structural alteration in synapses, via a reduction in cholesterol level [80]. In addition, the main SV clustering protein synapsin can affect cholesterol content in microdomains, promoting lipid raft formation [81].

**Figure 1:**
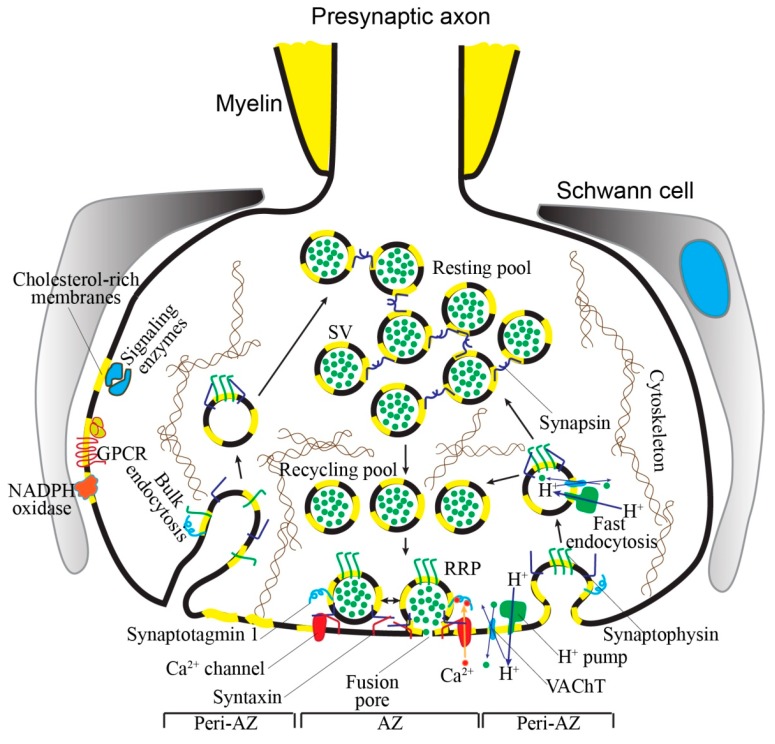
Putative role of cholesterol in presynaptic processes. Cholesterol organizes microdomains in presynaptic membrane and SVs. Several proteins, essential for presynaptic function, reside in these microdomains, and/or directly bind with cholesterol. These interactions are involved in control of multiple aspects of SV cycle that guarantees the maintenance of neurotransmitter release. Additionally, numerous presynaptic G-protein coupled receptors (GPCRs) and signaling enzymes (e.g., protein kinases and small GTPases), as well as a ROS-generating enzyme (NADPH oxidase), which regulates the steps of the SV cycle, could be located in cholesterol-rich microdomains. SV exocytosis occurs due to fusion of SVs from ready-releasable pool (RRP) with presynaptic membrane in AZ region. Under condition of moderate motor nerve activity, replenishment of RRP is mediated by delivery of SVs from recycling pool. After exocytosis, these SVs are able to rapidly recover through fast endocytotic mechanism, refill with ACh (green circles), and repeatedly participate in neurotransmitter release. Intense activation of motor neuron can led to mobilization of SVs from resting (reserve) pool to the exocytotic sites. After massive exocytosis, the resting pool is replenished by mainly slow endocytotic route (bulk endocytosis) associated with formation of endosome-like structures in NMJs. The cytoskeleton can contribute to spatial organization of both SV pools and routes for SV traffic. Key events, where the presence of cholesterol is required, are SV exocytosis (fusion pore formation, Ca^2+^ triggering step), endocytosis (vesicular protein holding in cluster), SV refilling with ACh (formation of proton gradient, exchange ACh and proton), SV clusterization (SV interconnections by synapsins), limitation of ACh leakage via spontaneous (clamping of signaling enzymes activity and ROS production), and nonquantal (decrease in activity of vesicular ACh transporter and H^+^-pump in presynaptic membrane) secretion. Please, for details, see Section 3 and Section 5.

Thus, changes in cholesterol levels can affect triggering exocytosis by Ca^2+^ (via Ca^2+^ channel and synaptotagmin), SV fusion (syntaxin) and endocytosis (synaptophysin), vesicle refilling with neurotransmitter (neurotransmitter transporters, H^+^ pump), and clusterization of SV (synapsin). Of course, changes in intracellular signaling molecules (e.g., phospholipases, protein kinases, and small GTPases) could mediate effects of cholesterol on synaptic transmission. Putative cholesterol-dependent steps in presynaptic vesicular cycle and cholesterol-sensitive proteins are shown in Figure 1 and Table 1.

AChE mainly resides in synaptic cleft, but the pool of AChE is located in association with lipid rafts through a GPI anchor [82]. Close distribution of raft-linked AChE to plasma membrane enriched with the receptors (e.g., M3 muscarinic receptors) could be important for control local levels of ACh near the receptors [83]. Accordingly, presence of cholesterol-rich microdomains may control sensitivity of feedback mechanisms which regulate ACh release from nerve terminals. Moreover, cholesterol-rich microdomains seem to be an important for development of depressant effect of ATP (cotransmitter of ACh) on neurotransmitter release at the frog NMJ. This effect is realized via P2Y12 receptors and ROS production by NADPH oxidase and it is reduced and largely delayed by cholesterol depletion [84]. In Drosophila NMJs, lipid rafts are required for presynaptic growth promoting effect of mannosyl glucosylceramide, which facilitates presynaptic Wnt1/Wingless signaling [85]. Thus, cholesterol is important component for coordination of receptor-dependent signaling, which regulates both neurotransmitter release and presynaptic bouton formation.

## 6. Cholesterol–Na,K-ATPase Interactions

Maintaining a steady state level of the RMP of muscle fibers is essential for the normal functioning of skeletal muscle. Membrane depolarization leads to a decrease in EPP amplitude, as well as inactivation of sodium channels and suppression of membrane excitability [1,6]. A smaller EPP amplitude and higher the AP generation threshold reduce safety factor at the NMJ. Among different mechanisms involved in maintaining skeletal muscle electrogenesis and contractile function, the activity of Na,K-ATPase plays a crucial role [86,87]. This transport system, discovered by Dr. J. Skou [88] (Nobel Prize in Chemistry, 1997), is an ubiquitous transmembrane protein that functions as a Na,K pump. Na,K-ATPase catalyzes the active transport of K^+^ into and Na^+^ out of the cell, thereby maintaining the steep Na^+^ and K^+^ gradients that provide electrical excitability and the driving force for many other transport processes [86,87,89,90,91]. Na,K-ATPase is critically important for excitability, electrogenesis, and contractility of skeletal muscles, which contain the main pool of the whole body Na,K-ATPase. The density of the distribution of Na,K-ATPase molecules in the sarcolemma is extremely high and ranges from 1000 to 3350/μm^2^ [86].

The influence of cholesterol on transmembrane protein (receptors, ion channels, transporters) function and properties is well established [8,9,92,93,94]. The function of Na,K-ATPase is also regulated by the lipid environment and strongly depends on membrane cholesterol content [93]. This regulation is realized via direct protein–lipid interactions or by influencing physical properties of the lipid bilayer [8,93,95].

Na,K-ATPase is composed of α catalytic and β glycoprotein subunits. Four isoforms of the α subunit are known to exist in tissues of vertebrates. It is generally accepted that the ubiquitous α1 isoform plays the main housekeeping role while the other isoforms expressing in a cell- and tissue-specific manner possess additional regulatory functions [89,90,96,97]. In skeletal muscles, the α1 and α2 isoforms of α subunit are coexpressed and the α2 Na,K-ATPase isozyme is predominant in adult skeletal muscle [98,99]. Distinct isoform-specific functions of Na,K-ATPase α1 and α2 isozyme in skeletal muscle are proposed [100,101,102,103,104]. The α2 Na,K-ATPase isozyme is specifically regulated by muscle use and enables working muscles to maintain excitability, contraction and resistance to fatigue [100,101,102,103]. Two main pools of the α2 Na,K-ATPase exist: the majority of α2 isozyme is expressed in the interior transverse tubule membranes [102], which are highly enriched in sphingomyelin and cholesterol compared to the surface sarcolemma [24]. The smaller α2 Na,K-ATPase isozyme pool is localized to the junctional (endplate) membrane [100,103], where cholesterol and lipid rafts serve as a potential signaling platform for nAChRs clustering [105,106]. Notably, these distinct α2 Na,K-ATPase membrane pools are regulated differently [103]. Both these α2 Na,K-ATPase membrane pools display the loss of electrogenic activity in response to hindlimb suspension (HS). However, the extrajunctional pool depends strongly on muscle disuse, and even the increased protein and mRNA content as well as enhanced α2 Na,K-ATPase membrane abundance after 12 h of HS cannot counteract this sustained inhibition. In contrast, additional factors (possibly circulating factors related to HS) may regulate the junctional α2 Na,K-ATPase pool that is able to recover during HS. Notably, acute, low intensity muscle workload restores functioning of both pools [103].

Subcellular compartmentalization is one of the basic principles of cellular organization [107,108]. A certain pool of Na,K-ATPase is localized in specialized lipid microdomains of the membrane—caveolae—where it forms regulatory multimolecular complexes and performs new functions, in particular, signal transduction [109,110,111,112,113]. As regulators of protein functions in caveolae and planar rafts may be the lipids themselves, including cholesterol. It was shown that cholesterol interacts with caveolins, principal components of caveolar membranes. Presumably, the α1 isoform of Na,K-ATPase contains binding sites for caveolin-1 located near the M1 and M10 transmembrane domains and interacted with the N-terminus of caveolin-1 [112,114,115]. Notably, caveolin-1 is involved in regulation of intracellular cholesterol traffic [116]. In experiments on cell lines, it has been shown that α1 Na,K-ATPase, through interaction with caveolin-1, participates in the distribution of cholesterol between intracellular membranes and the plasma membrane. On the one hand, the impaired expression of α1 Na,K-ATPase affects the formation of caveolae, cholesterol synthesis, and its traffic [117]. On the other hand, cholesterol itself is involved in the regulation of Na,K-ATPase. Thus, the reduction of cholesterol level in the plasma membrane stimulates endocytosis and degradation of α1 Na,K-ATPase via Src- and ubiquitin-dependent regulatory pathways [118]. The data obtained on the cell lines suggest the possibility of mutual regulation between cholesterol and α1 Na,K-ATPase which is carried out with the participation of caveolin-1.

In skeletal muscle, MβCD-induced partial membrane cholesterol removal selectively decreases the α2 Na,K-ATPase isozyme electrogenic activity without changing the α1 isozyme activity [119]. A similar specific dysfunction of the α2 Na,K-ATPase isozyme is also observed under the conditions of partial loss of membrane cholesterol in response to skeletal muscle motor unloading (disuse) [120]. Conversely, specific inhibition of the α2 Na,K-ATPase activity by ouabain induces a disturbance of lipid rafts due to partial cholesterol loss [120]. Collectively, these findings, obtained in alive skeletal muscles, suggest the reciprocal interactions between membrane cholesterol and the α2 Na,K-ATPase activity. It is important to note that the listed changes were most pronounced in the junctional membrane region [119,120].

While the molecular mechanisms of Na,K-ATPase regulation by surrounding lipids is being studied [93,95,121], little is known on how α2 Na,K-ATPase may regulate lipid raft stability. For example, this regulation may be mediated by α2 Na,K-ATPase ouabain receptor site. Notably, ouabain inhibits Na,K-ATPase transport by stabilizing the enzyme in an E2 conformation. Three specific lipid/Na,K-ATPase interactions are proposed that either stabilize the protein or stimulate/inhibit Na,K-ATPase activity, with separate binding sites and distinct kinetic mechanisms. Both stimulatory and inhibitory lipid interactions poise the conformational equilibrium toward the E2 state [121]. Altogether, these findings lead us to propose that reciprocal interactions between cholesterol and α2 Na,K-ATPase is more favored in the E2 enzyme conformation.

Regulation of α2 Na,K-ATPase is also specifically determined by its less stable integration into the lipid membrane compared with other α subunit isoforms. When studying Na,K-ATPase using *Pichia pastoris* yeast as an expression system, lipids, including cholesterol, have been shown to play an important role in stabilizing the Na,K-ATPase in the plasma membrane, and in an isoform-specific manner. Thus, a number of altering factors (heating, the use of detergents) predominantly affect the stability of insertion of the α2 Na,K-ATPase into the membrane compared to the α1 and α3 isoforms [122]. It is assumed that the relatively lower stability of the α2 isoform is due to the peculiarities of its transmembrane domains M8, M9, and M10, which are responsible for interaction with phospholipids, as well as weaker association with protein FXYD1 (phospholemman, an auxiliary regulatory subunit of the Na,K-ATPase) [123].

In sum, in skeletal muscle, the α1 Na,K-ATPase isozyme is functionally more stable compared with the α2 isozyme, whose adaptive plasticity is determined by specific localization and regulation of different enzyme pools and functional interactions with molecular environment including cholesterol.

## 7. Cholesterol–nAChR Interactions

Localization of neurotransmitter receptors in lipid microdomains and their interaction with cholesterol play an important role in the synaptic function and its plasticity, as well as in the development of a number of neurodegenerative diseases [54,124,125,126]. One of the important effects of the lipid environment is a change in receptor kinetics and their affinity for specific ligands. These changes can be explained both by the modulating effect of cholesterol due to its direct interaction with receptors, and by changing the properties of the membrane lipid bilayer [92,127,128,129].

**Figure 2:**
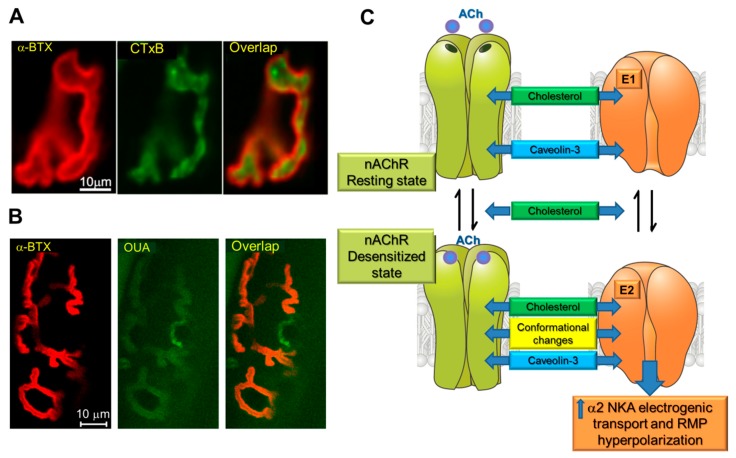
Cholesterol as a part of nAChR/α2Na,K-ATPase multimolecular regulatory complex. (**A**) The nAChRs and lipid rafts reside at the endplate region. A single endplate of rat soleus muscle was dual-labeled with α-BTX (nAChRs, red channel) and fluorescent cholera toxin B subunit to stain lipid rafts (CTxB, green channel). Overlap (orange channel). (**B**) The nAChR and the α2 Na,K-ATPase colocalization at the muscle endplate. A single endplate of mouse extensor digitorum longus muscle was dual-labeled with α-BTX (nAChRs, red channel) and BODIPY-conjugated ouabain (α2 Na,K-ATPase, green channel). Overlap (orange channel). Scale bars, 10 μm. (**C**) Hypothetical scheme of nAChR/α2Na,K-ATPase/Caveolin-3/Cholesterol interactions stimulating electrogenic active transport to hyperpolarize the resting membrane potential (RMP). Modified from Petrov et al. (2017) ref. [120] (A) and Heiny et al. (2010) ref. [100] (B).

nAChRs, as well as Na,K-ATPase, are integral membrane proteins that play key roles in membrane excitation. Role of cholesterol in the endplate membrane integrity is poorly understood due to deficit of ex vivo and in vivo studies. Both cholesterol and the nAChRs are localized at the junctional region (Figure 2A) and lipid rafts serve as a signaling platform for nAChRs clustering [105,106]. Previous investigations have demonstrated a direct molecular interaction between cholesterol and the nAChRs [92,130,131,132]. Surrounding lipids influence nAChRs kinetic mechanisms and cholesterol is critical for the channel function [129].

Cell studies suggest that peripheral membrane proteins, like rapsyn, contribute to organization of nAChRs cluster (prototype of postsynaptic nAChRs cluster) and cholesterol rich-microdomains are required for intracellular sorting and targeting of the nAChRs and rapsyn into the plasma membranes [133,134]. Also, lipid rafts and cholesterol content could be essential in the initial clustering and later stabilization of nAChRs clusters and organization signaling complex, including nAChRs, rapsyn, MuSK, and Src-family kinases [106,135,136]. Lipid rafts may regulate nAChRs clustering by facilitating the agrin/MuSK signaling and the interaction between the nAChRs and rapsyn; conversely, membrane cholesterol depletion inhibits nAChRs cluster formation [105]. Also, membrane cholesterol depletion disturbs the sarcolemma distribution of β-dystroglycan and its interaction with dystrophin [137], a key protein of the endplate integrity. Cholesterol stabilized nAChRs cluster in denervated muscle and could trigger maturation of nerve sprout-elicited nAChRs cluster into a “pretzel” shape [90]. An actin polymerization-dependent mechanism could facilitate lipid raft coalescence and thereby the formation of large nAChRs clusters [138].

Prolonged nerve activity and other conditions such as myopathy led to membrane depolarization. This depolarization causes inactivation of the sodium channels (localized at the bottom of postsynaptic folds), which plays an essential role in the loss of muscle fiber excitability [5]. The reduction of the excitability is most severe for the junctional region of the sarcolemma, where the local EPP is transformed into propagating AP.

Membrane depolarization increases the threshold of muscle AP generation and reduces the safety factor of neuromuscular transmission; hyperpolarization has the opposite effects [2]. In this regard, the fact that the junctional membrane of mammals is hyperpolarized by 2–4 mV compared to the extrajunctional region of the sarcolemma is especially important. Presumably, this local hyperpolarization resulted from the activation of Na,K-ATPase by nonhydrolyzed ACh, which is constantly present in the synaptic cleft in nanomolar concentration even with active AChE [139,140]. This residual ACh remains in the synaptic cleft for some time following quantal transmitter release, and also appears due to nonquantal ACh release. The physiological consequence of a local junctional membrane hyperpolarization is expected to be more effective muscle excitation and neuromuscular transmission.

A regulatory mechanism whereby nAChR and α2 Na,K-ATPase functionally and molecularly interact to modulate the RMP of skeletal muscle was identified [96,100,141]. The nAChR/Na,K-ATPase reciprocal interactions were demonstrated in a purified membrane preparation from *Torpedo californica*, enriched by nAChRs and Na,K-ATPase [100,141]. In this preparation, specific ligand binding to the nAChRs modulates specific ligand binding to the Na,K-ATPase and vice versa, suggesting the direct molecular interaction between these two proteins. Notably, similar reciprocal interaction between nAChR of neuronal type and Na,K-ATPase was further confirmed in an insect nervous system [142].

Moreover, the α2 Na,K-ATPase isozyme is enriched in junctional membrane region where it colocalizes with the nAChRs (Figure 2B). These proteins coimmunoprecipitate with each other, as well as with phospholemman (FXYD1 protein) and caveolin-3 [100]. Caveolin-3 is enriched at the junctional region where it co-localized with the nAChRs and promotes their clustering in the endplate membrane. The α subunit of the nAChR has a putative caveolin-binding motif and a lack of caveolin-3 inhibits nAChR clustering [143]. Also, the caveolin/Na,K-ATPase interactions are well-documented [112,114]. Since caveolin-3 is associated with caveolae in fully differentiated skeletal muscles [144], the nAChR/α2 Na,K-ATPase interaction likely takes place within caveolae [100].

In this interaction, the binding of ACh at nanomolar concentrations to the nAChRs stimulates electrogenic transport by the α2 Na,K-ATPase isozyme, causing a local junctional membrane hyperpolarization. Notably, the nAChRs oscillate between resting (micromolar affinity for ACh), open or desensitized (nonconducting state with nanomolar apparent affinity for ACh) conformations [145,146]. Micromolar concentrations of ACh promote channel opening following by spontaneous transitions into the desensitized state. Desensitization of the nAChRs can also occur without channel opening and is favored by prolonged exposure to low concentrations of agonist. Moreover, a number of facts suggest that it is the conformational change to desensitized state of the nAChRs that is responsible for the interaction with the α2 Na,K-ATPase [96,100]. Cholesterol and other lipids influence the rates of transitions between different nAChRs conformational states and a “conformational selection” model proposed that cholesterol modulate the equilibrium between resting and desensitized states [129]. In addition, sarcolemma cholesterol specifically contributes to maintaining endplate electrogenesis and cholesterol depletion by MβCD selectively decreases the α2 Na,K-ATPase isozyme electrogenic activity and eliminates local hyperpolarization [119] suggesting the involvement of cholesterol in formation and function of the nAChR/α2 Na,K-ATPase molecular complex.

Collectively, these findings suggest a mechanism by which the nAChRs in a nonconducting, desensitized state, with high apparent affinity for ACh, directly interacts with the α2 Na,K-ATPase to stimulate electrogenic active transport. The interaction utilizes a membrane-associated regulatory complex that includes the nAChR, the α2 Na,K-ATPase, FXYD1, caveolin-3 and cholesterol and is responsible for maintaining RMP and muscle excitability during intensive muscle use (Figure 2C).

## 8. Cholesterol and Motor Dysfunction

Physical exercise is extremely important to ensure a healthy life and, particularly, is essential for lipids metabolism [147]. While the impact of cholesterol and lipid rafts in pathogenesis of neurodegenerative disorders is well documented [54,124,125,126] much less is known about the role of cholesterol in neuromuscular disorders.

ALS is known to have a severe dysfunction of NMJs. Hence, ALS mouse model is an extensively studied model for NMJ diseases. The progression of ALS could be attributed to the alterations in cholesterol metabolism. High levels of plasma cholesterol and low density lipoprotein have neuroprotective effects in ALS patients, whereas inhibition of cholesterol synthesis with statins aggravates ALS progression [148,149,150]. Excess cholesterol could facilitate production of oxysterols which are ligands for liver X receptors (LXR). These receptors are expressed in skeletal muscles and the number of NMJs reduces in LXRβ-deficient mice [151]. Moreover, ablation of LXRβ led to an ALS-like pathology [152,153] and polymorphism of LXRs significantly affects ALS phenotype [154]. Stimulation of LXRs by oxysterols may decrease inflammation, increase antioxidant defense and affect lipid metabolism, thereby reducing the muscle damage [29]. However, certain oxysterols can have toxic effects. Notably, the plasma levels of 25HC were markedly increased in ALS patients and correlated with the disease severity [155]. One of the main sources of 25HC is mast cells and macrophages that may come into contact with degenerating NMJs, thereby accelerating axonopathy in the SOD1^G93A^ rats [156]. Lipid raft alterations can contribute to ALS progression. Decrease in lipid raft scaffold protein caveolin-1 was found in skeletal muscle of SOD1^G93A^ mice [157]. Additionally, omics analysis suggested that changes in abundance of numerous protein-residents of lipid rafts occur in spinal cord of ALS model mice [158]. In diaphragm, staining with lipid raft marker showed the presence of two populations of NMJs in SOD1^G93A^ mice containing less and more lipid rafts [39]. This may reflect the co-occurrence of opposite processes: a progressive NMJ dysfunction and a compensatory enhancement of synaptic function in the remaining NMJs.

The latest data indicate the leading role of motor activity in maintaining the level of membrane cholesterol. Recently, it has been shown that increased plasma lipid levels exacerbate muscle pathology in the mdx mouse model of Duchenne muscular dystrophy [159]. Another neuromuscular disorder is dysferlinopathy, induced by a deficiency of dysferlin protein. Dysferlin plays a key role in the multimolecular complex responsible for the repair of the integrity of sarcolemma during contractile activity. Dysferlin-deficient Bla/J mice (one of the models of dysferlinopathy) are also characterized by impaired lipid metabolism [160]. Notably, both mdx and Bla/J mice models are characterized by membrane depolarization resulting from lowered Na,K-ATPase electrogenic activity [6,161]. Dysferlin is a membrane-associated protein implicated in vesicle fusion, trafficking, and membrane resealing. Loss of dysferlin has a wide range of implications, such as decrease in membrane integrity, disturbances of the dynamics of membrane-associated molecules, Ca^2+^ dysregulation, Ca^2+^-induced proteolysis, and oxidative stress [162,163]. Together, these processes contribute to increased levels of necrosis and inflammation and result in the loss of motility. Furthermore, dysferlin-deficient muscles demonstrated an impaired glucose and lipid metabolism. Progressive adipocyte replacement and accumulation of lipid droplets within dysferlin-deficient myofibers [160] as well as extramyocellular lipid deposition [164,165] were observed. New evidence suggests that plasma membrane cholesterol accumulation in mice fed on a western high-fat diet may contribute by damaging the cortical F-actin structure that is essential for insulin-regulated GLUT4 translocation and glucose transport. It was interesting to note that exercise training has a preventive effect [166]. Also, elevated plasma cholesterol levels correlated with muscular pathology and can aggravate muscle fiber damage in dysferlinopathies [167]. In sum, such lipid abnormalities accompanied by fundamental metabolic disturbance suggest additional progressive decline of muscle function in dysferlinopathies.

Another muscular protein whose function could be linked with cholesterol availability is a member of the synaptophysin family, Mitsugumin 29, which contains cholesterol-binding MARVEL domain. Mitsugumin 29 is specifically located in the triad junction of muscle fibers and important for T-tubule formation, efficient excitation–contraction coupling, Ca^2+^ signaling, and resistance to fatigue [168,169,170]. Decreased expression of Mitsugumin 29 which leads to disturbance of triad junction structure and Ca^2+^ signaling could be partially linked with contractile dysfunction during muscle aging [171]. Similarly, cholesterol depletion affecting T-tubules reduced L-type Ca^2+^ current in freshly isolated fetal skeletal muscle cells [172]. Interestingly, a low-frequency coding variant in the gene encoding Mitsugumin 29 was associated with morbid obesity, suggesting a link between Mitsugumin 29 and lipid metabolism [173].

NMJ ultrastructure undergoes continual morphological remodeling in response to changes in the pattern of motor activity. Reduced activity (denervation, injury, bed rest and other form of disuse, microgravity at spaceflight, muscle diseases, and aging) triggers alterations in NMJ stability and integrity [174,175,176]. Even acute disuse (6–12 h of HS) disrupts plasma membrane lipid-ordered rafts in sarcolemma of rat soleus muscle [120]. This disturbance is accompanied by membrane depolarization due to loss of the α2 Na,K-ATPase electrogenic activity [103,177]. The greatest disturbances are observed at the junctional membrane region [103]. Moreover, specific inhibition of α2 Na,K-ATPase by 1 μM ouabain induces a disturbance of lipid rafts similar to that induced by disuse alone [120]. In both cases, lipid rafts were able to recover with cholesterol supplementation, suggesting that disturbance results from cholesterol loss. Repetitive nerve stimulation also restored both α2 Na,K-ATPase electrogenic activity and lipid rafts integrity. These findings suggest that the stability of lipid rafts is subject to regulation by skeletal muscle motor activity [120]. Intriguingly, important role of sphingolipids (including ceramide) in regulation of skeletal muscle function in health and disease is known [178] and it was shown that acute HS-induced cholesterol loss is accompanied by ceramide accumulation [179]. These reciprocal ceramide/cholesterol changes may reflect the ability of an excess of ceramide to displace cholesterol from sarcolemma [180].

Notably, adenosine monophosphate-activated protein kinase (AMPK) is an energy-sensing enzyme that is known to be a key regulator of glucose, lipid, and protein metabolism in skeletal muscle [181]. Particularly, AMPK has been implicated in control of sarcolemma cholesterol levels [166,182]. In addition, AMPK and AMPK-activated autophagy have been implicated in NMJ remodeling [183,184,185]. Lowered contractile activity should provide an accumulation of phosphorylated high-energy phosphates. Consistent with this, decreased phosphorylation of AMPK during 6–24 h of HS was demonstrated in rat soleus muscle [186,187,188].

These results provide evidence to suggest that the ordering of lipid rafts strongly depends on motor nerve input and may involve interactions with the α2 Na,K-ATPase and AMPK. Lipid raft disturbance, accompanied by the loss of α2 Na,K-ATPase activity and decreased phosphorylation of AMPK, are among the earliest remodeling events induced by skeletal muscle disuse. However, we should note that the effects of prolonged immobilization or unloading regarding skeletal muscle membrane remain poorly understood. Continuous membrane stretch can modulate the activity of channels and hence the dynamics of a variety of molecules incorporated into the lipid bilayer. Accordingly, stretch-induced lipid stress may trigger membrane remodeling including changes in cholesterol distribution. Thus, the question of whether membrane cholesterol disturbance results from muscle inactivity per se, or mediated by sarcolemma stretching, remains open.

## 9. Conclusions

**Figure 3:**
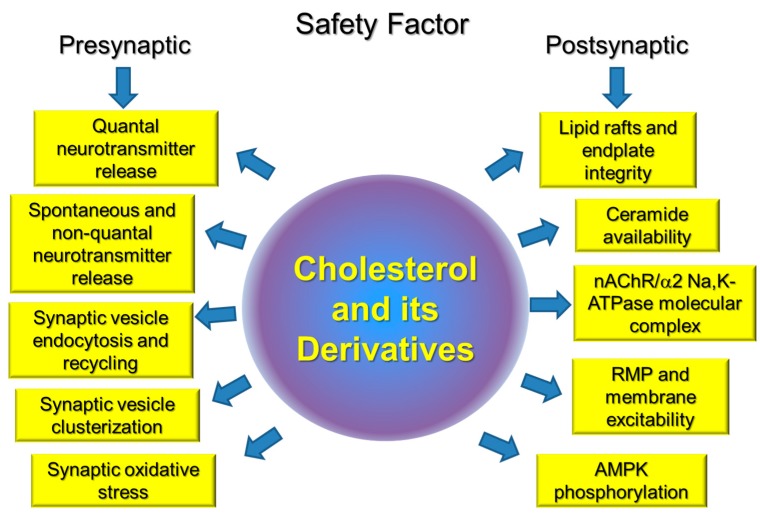
Key pre- and postsynaptic points that cross-talked with plasma membrane cholesterol and are responsible for the safety factor at the neuromuscular junction (NMJ).

Cholesterol, as important component of cell membranes, plays a crucial role in segregation of plasma membrane lipid phases and is essential to maintain membrane fluidity, curvature, ion channel and transporter functions, compartmentalization, and signaling. Cholesterol is specifically important for synaptic function, and a link between impaired cholesterol metabolism and neurodegenerative disorders is well recognized. However, much less is known about the role of cholesterol in peripheral neuromuscular transmission and corresponding disorders. Our review is the first attempt to summarize known experimental data on the role of cholesterol and its derivatives in the NMJ in health and some motor dysfunctions. Accumulated novel date indicates that cholesterol is included in all key pre- and postsynaptic processes on which the safety factor at the NMJ depends. Moreover, an imbalance of cholesterol homeostasis accompanied by membrane cholesterol changes can modulates these steps and disruption of lipid rafts is an early event in the disuse-induced muscle atrophy. Possible cholesterol-dependent steps, essential for maintaining safety factor for the neuromuscular transmission, are summarized in Figure 3. Future studies will be required to identify the precise molecular interactions between cholesterol and skeletal muscle motor activity.

## Figures and Tables

**Table 1:** Some potential interactions between cholesterol and proteins involved in regulation of neurotransmitter release from motor nerve terminals.

Protein	Role in Neuromuscular Transmission	Potential Role of Interaction with Cholesterol	Ref.
P2Y12 receptor	Inhibition of ACh release	Acceleration of the downstream receptor signaling	[84]
Ca^2+^ channels (N, L, P/Q types)	Triggering SV exocytosis in response to AP	Clusterization of channels near exocytotic sites, thereby facilitating exocytosis	[67,68,69]
NADPH oxidase (ROS generating enzyme)	Regulation of AP-evoked and spontaneous ACh release	Limitation of background activity	[41,84]
Proton pump	Formation of H^+^ gradient necessary for SV filling with ACh; regulation of SV size	Regulation of precise location and potentiation of H^+^ transport function	[74,75]
Signaling enzymes (protein kinases A/C)	Control of neurotransmitter release	Limitation of background activity of the protein kinases	[42,47]
Synapsin	Clusterization of SVs in pools	Lipid raft organization in SV membranes	[81]
Synaptophysin	Regulation of exo- and endocytosis	Induction of SV curvature during endocytosis	[52]
Synaptotagmin 1	A major Ca^2+^ sensor for neurotransmitter release	Location in lipid rafts and precise distribution in presynaptic membrane	[51,70]
Syntaxin	Component of SNARE complex mediated SV fusion	Clusterization in membrane; activity-dependent redistribution in lipid rafts	[76,77]
Vesicular ACh transporter	Uptake of ACh into SV, nonvesicular ACh release	Precise location in SV membranes and regulation of activity	[36]

## Abbreviations

α-BTX	rhodamine-conjugated α-bungarotoxin
ACh	acetylcholine
AChE	acetylcholinesterase
ALS	amyotrophic lateral sclerosis
AMPK	5′ adenosine monophosphate-activated protein kinase
AP	action potential
ATP	adenosine triphosphate
AZs	active zones
5Ch3	5α-cholestan-3-one
CSP	cysteine string protein
CTxB	cholera toxin B subunit
EPP	endplate potential
GPCRs	G-protein coupled receptors
24HC	24S-hydroxycholesterol
HS	hindlimb suspension
LXR	liver X receptor
MβCD	methyl-β-cyclodextrin
MEPP	miniature endplate potential
nAChR	nicotinic acetylcholine receptor
NADPH	nicotinamide adenine dinucleotide phosphate
NMJ	neuromuscular junction
RMP	resting membrane potential
ROS	reactive oxygen species
SOD1^G93A^	superoxide dismutase 1
SV	synaptic vesicle
VGLUT	vesicular glutamate transporter

## References

[1] Wood S.J., Slater C.R. (2001). Safety factor at the neuromuscular junction. Prog. Neurobiol..

[2] Ruff R.L. (2011). Endplate contributions to the safety factor for neuromuscular transmission. Muscle Nerve.

[3] Ackermann F., Waites C.L., Garner C.C. (2015). Presynaptic active zones in invertebrates and vertebrates. EMBO Rep..

[4] Sanes J.S., Lichtman J.W. (1999). Development of the vertebrate neuromuscular junction. Annu. Rev. Neurosci..

[5] Filatov G.N., Pinter M.J., Rich M.M. (2005). Resting Potential-dependent Regulation of the Voltage Sensitivity of Sodium Channel Gating in Rat Skeletal Muscle In Vivo. J. Gen. Physiol..

[6] Miles M.T., Cottey E., Cottey A., Stefanski C., Carlson C.G. (2011). Reduced resting potentials in dystrophic (mdx) muscle fibers are secondary to NF-κB-dependent negative modulation of ouabain sensitive Na^+^-K^+^ pump activity. J. Neurosci..

[7] Serra A., Ruff R.L., Leigh R.J. (2012). Neuromuscular transmission failure in myasthenia gravis: Decrement of safety factor and susceptibility of extraocular muscles. Ann. N. Y. Acad. Sci..

[8] Grouleff J., Irudayam S.J., Skeby K.K., Schiøtt B. (2015). The influence of cholesterol on membrane protein structure, function, and dynamics studied by molecular dynamics simulations. Biochim. Biophys. Acta.

[9] Belani J.D. (2019). Chirality Effect on Cholesterol Modulation of Protein Function. Adv. Exp. Med. Biol..

[10] Oh H., Mohler E.R., Tian A., Baumgart T., Diamond S.L. (2009). Membrane cholesterol is a biomechanical regulator of neutrophil adhesion. Arterioscler. Thromb. Vasc. Biol..

[11] Chun Y.S., Oh H.G., Park M.K., Kim T.W., Chung S. (2013). Increasing Membrane Cholesterol Level Increases the Amyloidogenic Peptide by Enhancing the Expression of Phospholipase C. J. Neurodegener. Dis..

[12] Magarkar A., Dhawan V., Kallinteri P., Viitala T., Elmowafy M., Róg T., Bunker A. (2014). Cholesterol level affects surface charge of lipid membranes in saline solution. Sci. Rep..

[13] Amsalem M., Poilbout C., Ferracci G., Delmas P., Padilla F. (2018). Membrane cholesterol depletion as a trigger of Nav1.9 channel-mediated inflammatory pain. EMBO J..

[14] Meleleo D., Sblano C. (2019). Influence of cholesterol on human calcitonin channel formation. Possible role of sterol as molecular chaperone. AIMS Biophys..

[15] Arenas F., Garcia-Ruiz C., Fernandez-Checa J.C. (2017). Intracellular Cholesterol Trafficking and Impact in Neurodegeneration. Front. Mol. Neurosci..

[16] Saher G., Stumpf S.K. (2015). Cholesterol in myelin biogenesis and hypomyelinating disorders. Biochim. Biophys. Acta.

[17] Pfrieger F.W., Ungerer N. (2011). Cholesterol metabolism in neurons and astrocytes. Prog. Lipid Res..

[18] Funfschilling U., Jockusch W.J., Sivakumar N., Mobius W., Corthals K., Li S., Quintes S., Kim Y., Schaap I.A., Rhee J.S. (2012). Critical time window of neuronal cholesterol synthesis during neurite outgrowth. J. Neurosci..

[19] Petrov A.M., Kasimov M.R., Zefirov A.L. (2016). Brain Cholesterol Metabolism and Its Defects: Linkage to Neurodegenerative Diseases and Synaptic Dysfunction. Acta Nat..

[20] Egawa J., Pearn M.L., Lemkuil B.P., Patel P.M., Head B.P. (2016). Membrane lipid rafts and neurobiology: Age-related changes in membrane lipids and loss of neuronal function. J. Physiol..

[21] Comley L.H., Fuller H.R., Wishart T.M., Mutsaers C.A., Thomson D., Wright A.K., Ribchester R.R., Morris G.E., Parson S.H., Horsburgh K. (2011). ApoE isoform-specific regulation of regeneration in the peripheral nervous system. Hum. Mol. Genet..

[22] Choi H.Y., Liu Y., Tennert C., Sugiura Y., Karakatsani A., Kröger S., Johnson E.B., Hammer R.E., Lin W., Herz J. (2013). APP interacts with LRP4 and agrin to coordinate the development of the neuromuscular junction in mice. eLife.

[23] Yokoyama M., Seo T., Park T., Yagyu H., Hu Y., Son N.H., Augustus A.S., Vikramadithyan R.K., Ramakrishnan R., Pulawa L.K. (2007). Effects of lipoprotein lipase and statins on cholesterol uptake into heart and skeletal muscle. J. Lipid Res..

[24] Barrientos G., Sánchez-Aguilera P., Jaimovich E., Hidalgo C., Llanos P. (2017). Membrane Cholesterol in Skeletal Muscle: A Novel Player in Excitation-Contraction Coupling and Insulin Resistance. J. Diabetes Res..

[25] Norata G.D., Tibolla G., Catapano A.L. (2014). Statins and skeletal muscles toxicity: From clinical trials to everyday practice. Pharmacol. Res..

[26] Greenberg A.S., Coleman R.A., Kraemer F.B., McManaman J.L., Obin M.S., Puri V., Yan Q.W., Miyoshi H., Mashek D.G. (2011). The role of lipid droplets in metabolic disease in rodents and humans. J. Clin. Investig..

[27] Murphy R.C., Johnson K.M. (2008). Cholesterol, reactive oxygen species, and the formation of biologically active mediators. J. Biol. Chem..

[28] Archer A., Laurencikiene J., Ahmed O., Steffensen K.R., Parini P., Gustafsson J.A., Korach-André M. (2014). Skeletal muscle as a target of LXR agonist after long-term treatment: Focus on lipid homeostasis. Am. J. Physiol. Endocrinol. Metab..

[29] Webb R., Hughes M.G., Thomas A.W., Morris K. (2017). The Ability of Exercise-Associated Oxidative Stress to Trigger Redox-Sensitive Signalling Responses. Antioxidants.

[30] Rizzoli S.O. (2014). Synaptic vesicle recycling: Steps and principles. EMBO J..

[31] Rizzoli S.O., Betz W.J. (2005). Synaptic vesicle pools. Nat. Rev. Neurosci..

[32] Nakajima Y., Bridgman P.C. (1981). Absence of filipin-sterol complexes from the membranes of active zones and acetylcholine receptor aggregates at frog neuromuscular junctions. J. Cell. Biol..

[33] Ko C.P., Propst J.W. (1986). Absence of sterol-specific complexes at active zones of degenerating and regenerating frog neuromuscular junctions. J. Neurocytol..

[34] Zamir O., Charlton M.P. (2006). Cholesterol and synaptic transmitter release at crayfish neuromuscular junctions. J. Physiol..

[35] Petrov A.M., Kudryashova K.E., Odnoshivkina Y.G., Zefirov A.L. (2011). Cholesterol and lipid rafts in the plasma membrane of nerve terminal and membrane of synaptic vesicles. Neurochem. J..

[36] Petrov A.M., Naumenko N.V., Uzinskaya K.V., Giniatullin A.R., Urazaev A.K., Zefirov A.L. (2011). Increased non-quantal release of acetylcholine after inhibition of endocytosis by methyl-β-cyclodextrin: The role of vesicular acetylcholine transporter. Neuroscience.

[37] Kasimov M.R., Giniatullin A.R., Zefirov A.L., Petrov A.M. (2015). Effects of 5α-cholestan-3-one on the synaptic vesicle cycle at the mouse neuromuscular junction. Biochim. Biophys. Acta.

[38] Kasimov M.R., Zakyrjanova G.F., Giniatullin A.R., Zefirov A.L., Petrov A.M. (2016). Similar oxysterols may lead to opposite effects on synaptic transmission: Olesoxime versus 5 α-cholestan-3-one at the frog neuromuscular junction. Biochim. Biophys. Acta.

[39] Mukhutdinova K.A., Kasimov M.R., Giniatullin A.R., Zakyrjanova G.F., Petrov A.M. (2018). 24S-hydroxycholesterol suppresses neuromuscular transmission in SOD1(G93A) mice: A possible role of NO and lipid rafts. Mol. Cell. Neurosci..

[40] Petrov A.M., Kasimov M.R., Giniatullin A.R., Tarakanova O.I., Zefirov A.L. (2010). The role of cholesterol in the exo- and endocytosis of synaptic vesicles in frog motor nerve endings. Neurosci. Behav. Physiol..

[41] Petrov A.M., Yakovleva A.A., Zefirov A.L. (2014). Role of membrane cholesterol in spontaneous exocytosis at frog neuromuscular synapses: Reactive oxygen species-calcium interplay. J. Physiol..

[42] Petrov A.M., Zakyrjanova G.F., Yakovleva A.A., Zefirov A.L. (2015). Inhibition of protein kinase C affects on mode of synaptic vesicle exocytosis due to cholesterol depletion. Biochem. Biophys. Res. Commun..

[43] Tarakanova O.I., Petrov A.M., Zefirov A.L. (2011). The role of membrane cholesterol in neurotransmitter release from motor nerve terminals. Dokl. Biol. Sci..

[44] Rodrigues H.A., Lima R.F., Fonseca Mde C., Amaral E.A., Martinelli P.M., Naves L.A., Gomez M.V., Kushmerick C., Prado M.A., Guatimosim C. (2013). Membrane cholesterol regulates different modes of synaptic vesicle release and retrieval at the frog neuromuscular junction. Eur. J. Neurosci..

[45] Teixeira G., Vieira L.B., Gomez M.V., Guatimosim C. (2012). Cholesterol as a key player in the balance of evoked and spontaneous glutamate release in rat brain cortical synaptosomes. Neurochem. Int..

[46] Thyagarajan B., Potian J.G., McArdle J.J., Baskaran P. (2017). Perturbation to Cholesterol at the Neuromuscular Junction Confers Botulinum Neurotoxin A Sensitivity to Neonatal Mice. Toxicol. Sci..

[47] Smith A.J., Sugita S., Charlton M.P. (2010). Cholesterol-dependent kinase activity regulates transmitter release from cerebellar synapses. J. Neurosci..

[48] Takamori S., Holt M., Stenius K., Lemke E.A., Grønborg M., Riedel D., Urlaub H., Schenck S., Brügger B., Ringler P. (2006). Molecular anatomy of a trafficking organelle. Cell.

[49] Dason J.S., Smith A.J., Marin L., Charlton M.P. (2010). Vesicular sterols are essential for synaptic vesicle cycling. J. Neurosci..

[50] Yue H.Y., Xu J. (2015). Cholesterol regulates multiple forms of vesicle endocytosis at a mammalian central synapse. J. Neurochem..

[51] Dason J.S., Smith A.J., Marin L., Charlton M.P. (2014). Cholesterol and F-actin are required for clustering of recycling synaptic vesicle proteins in the presynaptic plasma membrane. J. Physiol..

[52] Thiele C., Hannah M.J., Fahrenholz F., Huttner W.B. (2000). Cholesterol binds to synaptophysin and is required for biogenesis of synaptic vesicles. Nat. Cell. Biol..

[53] Fewou S.N., Rupp A., Nickolay L.E., Carrick K., Greenshields K.N., Pediani J., Plomp J.J., Willison H.J. (2012). Anti-ganglioside antibody internalization attenuates motor nerve terminal injury in a mouse model of acute motor axonal neuropathy. J. Clin. Investig..

[54] Petrov A.M., Kasimov M.R., Zefirov A.L. (2017). Cholesterol in the Pathogenesis of Alzheimer’s, Parkinson’s Diseases and Autism: Link to Synaptic Dysfunction. Acta Nat..

[55] Testa G., Rossin D., Poli G., Biasi F., Leonarduzzi G. (2018). Implication of oxysterols in chronic inflammatory human diseases. Biochimie.

[56] Petrov A.M., Kasimov M.R., Giniatullin A.R., Zefirov A.L. (2014). Effects of Oxidation of Membrane Cholesterol on the Vesicle Cycle in Motor Nerve Terminals in the Frog Rana Ridibunda. Neurosci. Behav. Physiol..

[57] Kasimov M.R., Fatkhrakhmanova M.R., Mukhutdinova K.A., Petrov A.M. (2017). 24S-Hydroxycholesterol enhances synaptic vesicle cycling in the mouse neuromuscular junction: Implication of glutamate NMDA receptors and nitric oxide. Neuropharmacology.

[58] Xiao Y., Karam C., Yi J., Zhang L., Li X., Yoon D., Wang H., Dhakal K., Ramlow P., Yu T. (2018). ROS-related mitochondrial dysfunction in skeletal muscle of an ALS mouse model during the disease progression. Pharmacol. Res..

[59] Di Stasi A.M., Mallozzi C., Macchia G., Maura G., Petrucci T.C., Minetti M. (2002). Peroxynitrite affects exocytosis and SNARE complex formation and induces tyrosine nitration of synaptic proteins. J. Neurochem..

[60] Thomas S., Robitaille R. (2001). Differential frequency-dependent regulation of transmitter release by endogenous nitric oxide at the amphibian neuromuscular synapse. J. Neurosci..

[61] Robinson S.W., Bourgognon J.M., Spiers J.G., Breda C., Campesan S., Butcher A., Mallucci G.R., Dinsdale D., Morone N., Mistry R. (2018). Nitric oxide-mediated posttranslational modifications control neurotransmitter release by modulating complexin farnesylation and enhancing its clamping ability. PLoS Biol..

[62] DeBarber A.E., Sandlers Y., Pappu A.S., Merkens L.S., Duell P.B., Lear S.R., Erickson S.K., Steiner R.D. (2011). Profiling sterols in cerebrotendinous xanthomatosis: Utility of Girard derivatization and high resolution exact mass LC-ESI-MS(n) analysis. J. Chromatogr. B Anal. Technol. Biomed. Life Sci..

[63] Martin L.J. (2010). Olesoxime, a cholesterol-like neuroprotectant for the potential treatment of amyotrophic lateral sclerosis. IDrugs.

[64] Gouarné C., Tracz J., Paoli M.G., Deluca V., Seimandi M., Tardif G., Xilouri M., Stefanis L., Bordet T., Pruss R.M. (2015). Protective role of olesoxime against wild-type α-synuclein-induced toxicity in human neuronally differentiated SHSY-5Y cells. Br. J. Pharmacol..

[65] Magalon K., Le Grand M., El Waly B., Moulis M., Pruss R., Bordet T., Cayre M., Belenguer P., Carré M., Durbec P. (2016). Olesoxime favors oligodendrocyte differentiation through a functional interplay between mitochondria and microtubules. Neuropharmacology.

[66] Weber J.J., Ortiz Rios M.M., Riess O., Clemens L.E., Nguyen H.P. (2016). The calpain-suppressing effects of olesoxime in Huntington’s disease. Rare Dis..

[67] Taverna E., Saba E., Rowe J., Francolini M., Clementi F., Rosa P. (2004). Role of lipid microdomains in P/Q-type calcium channel (Cav2.1) clustering and function in presynaptic membranes. J. Biol. Chem..

[68] Thoreson W.B., Mercer A.J., Cork K.M., Szalewski R.J. (2013). Lateral mobility of L-type calcium channels in synaptic terminals of retinal bipolar cells. Mol. Vis..

[69] Ronzitti G., Bucci G., Emanuele M., Leo D., Sotnikova T.D., Mus L.V., Soubrane C.H., Dallas M.L., Thalhammer A., Cingolani L.A. (2014). Exogenous α-synuclein decreases raft partitioning of Cav2.2 channels inducing dopamine release. J. Neurosci..

[70] Lv J.H., He L., Sui S.F. (2008). Lipid rafts association of synaptotagmin I on synaptic vesicles. Biochemistry.

[71] Matthies H.J., Han Q., Shields A., Wright J., Moore J.L., Winder D.G., Galli A., Blakely R.D. (2009). Subcellular localization of the antidepressant-sensitive norepinephrine transporter. BMC Neurosci..

[72] De Juan-Sanz J., Núñez E., Zafra F., Berrocal M., Corbacho I., Ibáñez I., Arribas-González E., Marcos D., López-Corcuera B., Mata A.M. (2014). Presynaptic control of glycine transporter 2 (GlyT2) by physical and functional association with plasma membrane Ca^2+^-ATPase (PMCA) and Na^+^-Ca^2+^ exchanger (NCX). J. Biol. Chem..

[73] Rahbek-Clemmensen T., Lycas M.D., Erlendsson S., Eriksen J., Apuschkin M., Vilhardt F., Jørgensen T.N., Hansen F.H., Gether U. (2017). Super-resolution microscopy reveals functional organization of dopamine transporters into cholesterol and neuronal activity-dependent nanodomains. Nat. Commun..

[74] Yoshinaka K., Kumanogoh H., Nakamura S., Maekawa S. (2004). Identification of V-ATPase as a major component in the raft fraction prepared from the synaptic plasma membrane and the synaptic vesicle of rat brain. Neurosci. Lett..

[75] Lee J.S., Cho W.J., Shin L., Jena B.P. (2010). Involvement of cholesterol in synaptic vesicle swelling. Exp. Biol. Med..

[76] Murray D.H., Tamm L.K. (2011). Molecular mechanism of cholesterol- and polyphosphoinositide-mediated syntaxin clustering. Biochemistry.

[77] Gil C., Cubí R., Blasi J., Aguilera J. (2006). Synaptic proteins associate with a sub-set of lipid rafts when isolated from nerve endings at physiological temperature. Biochem. Biophys. Res. Commun..

[78] Cho W.J., Jeremic A., Jin H., Ren G., Jena B.P. (2007). Neuronal fusion pore assembly requires membrane cholesterol. Cell Biol. Int..

[79] Kreutzberger A.J., Kiessling V., Tamm L.K. (2015). High cholesterol obviates a prolonged hemifusion intermediate in fast SNARE-mediated membrane fusion. Biophys. J..

[80] Kyung J.W., Kim J.M., Lee W., Ha T.Y., Cha S.H., Chung K.H., Choi D.J., Jou I., Song W.K., Joe E.H. (2018). DJ-1 deficiency impairs synaptic vesicle endocytosis and reavailability at nerve terminals. Proc. Natl. Acad. Sci. USA.

[81] Kao H.T., Ryoo K., Lin A., Janoschka S.R., Augustine G.J., Porton B. (2017). Synapsins regulate brain-derived neurotrophic factor-mediated synaptic potentiation and axon elongation by acting on membrane rafts. Eur. J. Neurosci..

[82] Moral-Naranjo M.T., Montenegro M.F., Muñoz-Delgado E., Campoy F.J., Vidal C.J. (2010). The levels of both lipid rafts and raft-located acetylcholinesterase dimers increase in muscle of mice with muscular dystrophy by merosin deficiency. Biochim. Biophys. Acta.

[83] Montenegro M.F., Cabezas-Herrera J., Campoy F.J., Muñoz-Delgado E., Vidal C.J. (2017). Lipid rafts of mouse liver contain nonextended and extended acetylcholinesterase variants along with M3 muscarinic receptors. FASEB J..

[84] Giniatullin A., Petrov A., Giniatullin R. (2015). The involvement of P2Y12 receptors, NADPH oxidase, and lipid rafts in the action of extracellular ATP on synaptic transmission at the frog neuromuscular junction. Neuroscience.

[85] Huang Y., Huang S., Di Scala C., Wang Q., Wandall H.H., Fantini J., Zhang Y.Q. (2018). The glycosphingolipid MacCer promotes synaptic bouton formation in *Drosophila* by interacting with Wnt. eLife.

[86] Clausen T. (2003). Na^+^-K^+^ pump regulation and skeletal muscle contractility. Physiol. Rev..

[87] Clausen T. (2013). Quantification of Na^+^,K^+^ pumps and their transport rate in skeletal muscle: Functional significance. J. Gen. Physiol..

[88] Skou J.C. (1957). The influence of some cations on an adenosine triphosphatase from peripheral nerves. Biochim. Biophys. Acta.

[89] Blanco G., Mercer R.W. (1998). Isozymes of the Na-K-ATPase: Heterogeneity in structure, diversity in function. Am. J. Physiol..

[90] Mobasheri A., Avila J., Cózar-Castellano I., Brownleader M.D., Trevan M., Francis M.J., Lamb J.F., Martín-Vasallo P. (2000). Na^+^,K^+^-ATPase isozyme diversity; comparative biochemistry and physiological implications of novel functional interactions. Biosci. Rep..

[91] Sejersted O.M., Sjogaard G. (2000). Dynamics and consequences of potassium shifts in skeletal muscle and heart during exercise. Physiol. Rev..

[92] Levitan I., Singh D.K., Rosenhouse-Dantsker A. (2014). Cholesterol binding to ion channels. Front. Physiol..

[93] Cornelius F., Habeck M., Kanai R., Toyoshima C., Karlish S.J. (2015). General and specific lipid-protein interactions in Na,K-ATPase. Biochim. Biophys. Acta.

[94] Sibarov D.A., Poguzhelskaya E.E., Antonov S.M. (2018). Downregulation of calcium-dependent NMDA receptor desensitization by sodium-calcium exchangers: A role of membrane cholesterol. BMC Neurosci..

[95] Haviv H., Habeck M., Kanai R., Toyoshima C., Karlish S.J. (2013). Neutral phospholipids stimulate Na,K-ATPase activity: A specific lipid-protein interaction. J. Biol. Chem..

[96] Matchkov V.V., Krivoi I.I. (2016). Specialized functional diversity and interactions of the Na,K-ATPase. Front. Physiol..

[97] Clausen M.V., Hilbers F., Poulsen H. (2017). The Structure and Function of the Na,K-ATPase Isoforms in Health and Disease. Front. Physiol..

[98] Orlowski J., Lingrel J.B. (1988). Tissue-specific and developmental regulation of rat Na,K-ATPase catalytic α isoform and β subunit mRNAs. J. Biol. Chem..

[99] He S., Shelly D.A., Moseley A.E., James P.F., James J.H., Paul R.J., Lingrel J.B. (2001). The α1- and α2-isoforms of Na-K-ATPase play different roles in skeletal muscle contractility. Am. J. Physiol. Regul. Integr. Comp. Physiol..

[100] Heiny J.A., Kravtsova V.V., Mandel F., Radzyukevich T.L., Benziane B., Prokofiev A.V., Pedersen S.E., Chibalin A.V., Krivoi I.I. (2010). The nicotinic acetylcholine receptor and the Na,K-ATPase α2 isoform interact to regulate membrane electrogenesis in skeletal muscle. J. Biol. Chem..

[101] Radzyukevich T.L., Neumann J.C., Rindler T.N., Oshiro N., Goldhamer D.J., Lingrel J.B., Heiny J.A. (2013). Tissue-specific role of the Na,K-ATPase α2 isozyme in skeletal muscle. J. Biol. Chem..

[102] DiFranco M., Hakimjavadi H., Lingrel J.B., Heiny J.A. (2015). Na,K-ATPase α2 activity in mammalian skeletal muscle T-tubules is acutely stimulated by extracellular K^+^. J. Gen. Physiol..

[103] Kravtsova V.V., Petrov A.M., Matchkov V.V., Bouzinova E.V., Vasiliev A.N., Benziane B., Zefirov A.L., Chibalin A.V., Heiny J.A., Krivoi I.I. (2016). Distinct α2 Na,K-ATPase membrane pools are differently involved in early skeletal muscle remodeling during disuse. J. Gen. Physiol..

[104] Kutz L.C., Mukherji S.T., Wang X., Bryant A., Larre I., Heiny J.A., Lingrel J.B., Pierre S.V., Xie Z. (2018). Isoform-specific role of Na/K-ATPase α1 in skeletal muscle. Am. J. Physiol. Endocrinol. Metab..

[105] Zhu D., Xiong W.C., Mei L. (2006). Lipid rafts serve as a signaling platform for nicotinic acetylcholine receptor clustering. J. Neurosci..

[106] Willmann R., Pun S., Stallmach L., Sadasivam G., Santos A.F., Caroni P., Fuhrer C. (2006). Cholesterol and lipid microdomains stabilize the postsynapse at the neuromuscular junction. EMBO J..

[107] Saks V., Monge C., Guzun R. (2009). Philosophical basis and some historical aspects of systems biology: From Hegel to Noble—Applications for bioenergetic research. Int. J. Mol. Sci..

[108] Lingwood D., Simons K. (2010). Lipid rafts as a membrane-organizing principle. Science.

[109] Schoner W., Scheiner-Bobis G. (2007). Endogenous and exogenous cardiac glycosides and their mechanisms of action. Am. J. Cardiovasc. Drugs.

[110] Tian J., Xie Z. (2008). The Na-K-ATPase and calcium-signaling microdomains. Physiology.

[111] Lingrel J.B. (2010). The physiological significance of the cardiotonic steroid/ouabain-binding site of the Na,K-ATPase. Annu. Rev. Physiol..

[112] Morrill G.A., Kostellow A.B., Askari A. (2012). Caveolin-Na/K-ATPase interactions: Role of transmembrane topology in non-genomic steroid signal transduction. Steroids.

[113] Cui X., Xie Z. (2017). Protein Interaction and Na/K-ATPase-Mediated Signal Transduction. Molecules.

[114] Wang H., Haas M., Liang M., Cai T., Tian J., Li S., Xie Z. (2004). Ouabain assembles signaling cascades through the caveolar Na^+^/K^+^-ATPase. J. Biol. Chem..

[115] Cai T., Wang H., Chen Y., Liu L., Gunning W.T., Quintas L.E., Xie Z.J. (2008). Regulation of caveolin-1 membrane trafficking by the Na/K-ATPase. J. Cell Biol..

[116] Razani B., Woodman S.E., Lisanti M.P. (2002). Caveolae: From Cell Biology to Animal Physiology. Pharmacol. Rev..

[117] Chen Y., Cai T., Wang H., Li Z., Loreaux E., Lingrel J.B., Xie Z. (2009). Regulation of intracellular cholesterol distribution by Na/K-ATPase. J. Biol. Chem..

[118] Chen Y., Li X., Ye Q., Tian J., Jing R., Xie Z. (2011). Regulation of α1 Na/K-ATPase expression by cholesterol. J. Biol. Chem..

[119] Kravtsova V.V., Petrov A.M., Vasiliev A.N., Zefirov A.L., Krivoi I.I. (2015). Role of cholesterol in the maintenance of endplate electrogenesis in rat diaphragm. Bull. Exp. Biol. Med..

[120] Petrov A.M., Kravtsova V.V., Matchkov V.V., Vasiliev A.N., Zefirov A.L., Chibalin A.V., Heiny J.A., Krivoi I.I. (2017). Membrane lipid rafts are disturbed in the response of rat skeletal muscle to short-term disuse. Am. J. Physiol. Cell Physiol..

[121] Habeck M., Haviv H., Katz A., Kapri-Pardes E., Ayciriex S., Shevchenko A., Ogawa H., Toyoshima C., Karlish S.J. (2015). Stimulation, inhibition, or stabilization of Na,K-ATPase caused by specific lipid interactions at distinct sites. J. Biol. Chem..

[122] Lifshitz Y., Petrovich E., Haviv H., Goldshleger R., Tal D.M., Garty H., Karlish S.J.D. (2007). Purification of the human α2 isoform of Na,K-ATPase expressed in *Pichia pastoris*. Stabilization by lipids and FXYD1. Biochemistry.

[123] Kapri-Pardes E., Katz A., Haviv H., Mahmmoud Y., Ilan M., Khalfin-Penigel I., Carmeli S., Yarden O., Karlish S.J.D. (2011). Stabilization of the α2 isoform of Na,K-ATPase by mutations in a phospholipid binding pocket. J. Biol. Chem..

[124] Allen J.A., Halverson-Tamboli R.A., Rasenick M.M. (2007). Lipid raft microdomains and neurotransmitter signalling. Nat. Rev. Neurosci..

[125] Hicks D.A., Nalivaeva N.N., Turner A.J. (2012). Lipid rafts and Alzheimer’s disease: Protein-lipid interactions and perturbation of signaling. Front. Physiol..

[126] Sebastiao A.M., Colino-Oliveira M., Assaife-Lopes N., Dias R.B., Ribeiro J.A. (2013). Lipid rafts, synaptic transmission and plasticity: Impact in age-related neurodegenerative diseases. Neuropharmacology.

[127] Sooksawate T., Simmonds M.A. (2001). Effects of membrane cholesterol on the sensitivity of the GABAA receptor to GABA in acutely dissociated rat hippocampal neurones. Neuropharmacology.

[128] Eroglu C., Brugger B., Wieland F., Sinning I. (2003). Glutamate-binding affinity of *Drosophila* metabotropic glutamate receptor is modulated by association with lipid rafts. Proc. Natl. Acad. Sci. USA.

[129] Baenziger J.E., Domville J.A., Therien J.P.D. (2017). The Role of Cholesterol in the Activation of Nicotinic Acetylcholine Receptors. Curr. Top. Membr..

[130] Burger K., Gimpl G., Fahrenholz F. (2000). Regulation of receptor function by cholesterol. Cell. Mol. Life Sci..

[131] Brannigan G., Hénin J., Law R., Eckenhoff R., Klein M.L. (2008). Embedded cholesterol in the nicotinic acetylcholine receptor. Proc. Natl. Acad. Sci. USA.

[132] Barrantes F.J. (2016). Cholesterol and nicotinic acetylcholine receptor: An intimate nanometer-scale spatial relationship spanning the billion year time-scale. Biomed. Spectrosc. Imaging.

[133] Scher M.G., Bloch R.J. (1991). The lipid bilayer of acetylcholine receptor clusters of cultured rat myotubes is organized into morphologically distinct domains. Exp. Cell. Res..

[134] Marchand S., Devillers-Thiéry A., Pons S., Changeux J.P., Cartaud J. (2002). Rapsyn escorts the nicotinic acetylcholine receptor along the exocytic pathway via association with lipid rafts. J. Neurosci..

[135] Campagna J.A., Fallon J. (2006). Lipid rafts are involved in C95 (4,8) agrin fragment-induced acetylcholine receptor clustering. Neuroscience.

[136] Stetzkowski-Marden F., Gaus K., Recouvreur M., Cartaud A., Cartaud J. (2006). Agrin elicits membrane lipid condensation at sites of acetylcholine receptor clusters in C2C12 myotubes. J. Lipid Res..

[137] Vega-Moreno J., Tirado-Cortes A., Álvarez R., Irles C., Mas-Oliva J., Ortega A. (2012). Cholesterol depletion uncouples β-dystroglycans from discrete sarcolemmal domains, reducing the mechanical activity of skeletal muscle. Cell Physiol. Biochem..

[138] Pato C., Stetzkowski-Marden F., Gaus K., Recouvreur M., Cartaud A., Cartaud J. (2008). Role of lipid rafts in agrin-elicited acetylcholine receptor clustering. Chem. Biol. Interact..

[139] Nikolsky E.E., Zemkova H., Voronin V.A., Vyskocil F. (1994). Role of non-quantal acetylcholine release in surplus polarization of mouse diaphragm fibres at the endplate zone. J. Physiol..

[140] Vyskocil F., Malomouzh A.I., Nikolsky E.E. (2009). Non-quantal acetylcholine release at the neuromuscular junction. Physiol. Res..

[141] Krivoi I.I., Drabkina T.M., Kravtsova V.V., Vasiliev A.N., Eaton M.J., Skatchkov S.N., Mandel F. (2006). On the functional interaction between nicotinic acetylcholine receptor and Na^+^,K^+^-ATPase. Pflugers Arch..

[142] Bao H., Sun H., Xiao Y., Zhang Y., Wang X., Xu X., Liu Z., Fang J., Li Z. (2015). Functional interaction of nicotinic acetylcholine receptors and Na^+^/K^+^ ATPase from *Locusta migratoria manilensis* (Meyen). Sci. Rep..

[143] Hezel M., de Groat W.C., Galbiati F. (2010). Caveolin-3 promotes nicotinic acetylcholine receptor clustering and regulates neuromuscular junction activity. Mol. Biol. Cell.

[144] Galbiati F., Razani B., Lisanti M.P. (2001). Caveolae and caveolin-3 in muscular dystrophy. Trends Mol. Med..

[145] Prince R.J., Sine S.M. (1999). Acetylcholine and epibatidine binding to muscle acetylcholine receptors distinguish between concerted and uncoupled models. J. Biol. Chem..

[146] Mourot A., Rodrigo J., Kotzyba-Hibert F., Bertrand S., Bertrand D., Goeldner M. (2006). Probing the reorganization of the nicotinic acetylcholine receptor during desensitization by time-resolved covalent labeling using [^3^H]AC5, a photoactivatable agonist. Mol. Pharmacol..

[147] Radak Z., Suzuki K., Higuchi M., Balogh L., Boldogh I., Koltai E. (2016). Physical exercise, reactive oxygen species and neuroprotection. Free Radic. Biol. Med..

[148] Dupuis L., Corcia P., Fergani A., Gonzalez De Aguilar J.L., Bonnefont-Rousselot D., Bittar R., Seilhean D., Hauw J.J., Lacomblez L., Loeffler J.P. (2008). Dyslipidemia is a protective factor in amyotrophic lateral sclerosis. Neurology.

[149] Dorst J., Kuhnlein P., Hendrich C., Kassubek J., Sperfeld A.D., Ludolph A.C. (2011). Patients with elevated triglyceride and cholesterol serum levels have a prolonged survival in amyotrophic lateral sclerosis. J. Neurol..

[150] Zheng Z., Sheng L., Shang H. (2013). Statins and amyotrophic lateral sclerosis: A systematic review and meta-analysis. Amyotroph. Lateral. Scler. Frontotemporal. Degener..

[151] Bigini P., Steffensen K.R., Ferrario A., Diomede L., Ferrara G., Barbera S., Salzano S., Fumagalli E., Ghezzi P., Mennini T. (2010). Neuropathologic and biochemical changes during disease progression in liver X receptor beta^−/−^ mice, a model of adult neuron disease. J. Neuropathol. Exp. Neurol..

[152] Andersson S., Gustafsson N., Warner M., Gustafsson J.A. (2005). Inactivation of liver X receptor beta leads to adultonset motor neuron degeneration in male mice. Proc. Natl. Acad. Sci. USA.

[153] Kim H.J., Fan X., Gabbi C., Yakimchuk K., Parini P., Warner M., Gustafsson J.A. (2008). Liver X receptor beta (LXRbeta): A link between beta-sitosterol and amyotrophic lateral sclerosis-Parkinson’s dementia. Proc. Natl. Acad. Sci. USA.

[154] Mouzat K., Molinari N., Kantar J., Polge A., Corcia P., Couratier P., Clavelou P., Juntas-Morales R., Pageot N., Lobaccaro J.-A. (2018). Liver X Receptor Genes Variants Modulate ALS Phenotype. Mol. Neurobiol..

[155] Kim S.M., Noh M.Y., Kim H., Cheon S.Y., Lee K.M., Lee J., Cha E., Park K.S., Lee K.W., Sung J.J. (2017). 25-Hydroxycholesterol is involved in the pathogenesis of amyotrophic lateral sclerosis. Oncotarget.

[156] Trias E., Ibarburu S., Barreto-Núñez R., Varela V., Moura I.C., Dubreuil P., Hermine O., Beckman J.S., Barbeito L. (2017). Evidence for mast cells contributing to neuromuscular pathology in an inherited model of ALS. JCI Insight.

[157] Flis D.J., Dzik K., Kaczor J.J., Halon-Golabek M., Antosiewicz J., Wieckowski M.R., Ziolkowski W. (2018). Swim Training Modulates Skeletal Muscle Energy Metabolism, Oxidative Stress, and Mitochondrial Cholesterol Content in Amyotrophic Lateral Sclerosis Mice. Oxid. Med. Cell. Longev..

[158] Zhai J., Ström A.L., Kilty R., Venkatakrishnan P., White J., Everson W.V., Smart E.J., Zhu H. (2009). Proteomic characterization of lipid raft proteins in amyotrophic lateral sclerosis mouse spinal cord. FEBS J..

[159] Milad N., White Z., Tehrani A.Y., Sellers S., Rossi F.M.V., Bernatchez P. (2017). Increased plasma lipid levels exacerbate muscle pathology in the mdx mouse model of Duchenne muscular dystrophy. Skelet. Muscle.

[160] Grounds M.D., Terrill J.R., Radley-Crabb H.G., Robertson T., Papadimitriou J., Spuler S., Shavlakadze T. (2014). Lipid accumulation in dysferlin-deficient muscles. Am. J. Pathol..

[161] Kravtsova V.V., Timonina N.A., Zakir’yanova G.F., Sokolova A.V., Mikhailov V.M., Zefirov A.L., Krivoi I.I. (2018). The Structural and Functional Characteristics of the Motor End Plates of Dysferlin-Deficient Mice. Neurochem. J..

[162] Kerr J.P., Ward C.W., Bloch R.J. (2014). Dysferlin at transverse tubules regulates Ca^2+^ homeostasis in skeletal muscle. Front. Physiol..

[163] Demonbreun A.R., Allen M.V., Warner J.L., Barefield D.Y., Krishnan S., Swanson K.E., Earley J.U., McNally E.M. (2016). Enhanced Muscular Dystrophy from Loss of Dysferlin Is Accompanied by Impaired Annexin A6 Translocation after Sarcolemmal Disruption. Am. J. Pathol..

[164] Nagy N., Nonneman R.J., Llanga T., Dial C.F., Riddick N.V., Hampton T., Moy S.S., Lehtimäki K.K., Ahtoniemi T., Puoliväli J. (2017). Hip region muscular dystrophy and emergence of motor deficits in dysferlin-deficient Bla/J mice. Physiol. Rep..

[165] Llanga T., Nagy N., Conatser L., Dial C., Sutton R.B., Hirsch M.L. (2017). Structure-Based Designed Nano-Dysferlin Significantly Improves Dysferlinopathy in BLA/J Mice. Mol. Ther..

[166] Ambery A.G., Tackett L., Penque B.A., Brozinick J.T., Elmendorf J.S. (2017). Exercise training prevents skeletal muscle plasma membrane cholesterol accumulation, cortical actin filament loss, and insulin resistance in C57BL/6J mice fed a western-style high-fat diet. Physiol. Rep..

[167] Sellers S.L., Milad N., White Z., Pascoe C., Chan R., Payne G.W., Seow C., Rossi F., Seidman M.A., Bernatchez P. (2018). Increased nonHDL cholesterol levels cause muscle wasting and ambulatory dysfunction in the mouse model of LGMD2B. J. Lipid Res..

[168] Nagaraj R.Y., Nosek C.M., Brotto M.A., Nishi M., Takeshima H., Nosek T.M., Ma J. (2000). Increased susceptibility to fatigue of slow- and fast-twitch muscles from mice lacking the MG29 gene. Physiol. Genom..

[169] Brandt N.R., Franklin G., Brunschwig J.P., Caswell A.H. (2001). The role of mitsugumin 29 in transverse tubules of rabbit skeletal muscle. Arch. Biochem. Biophys..

[170] Brotto M.A., Nagaraj R.Y., Brotto L.S., Takeshima H., Ma J.J., Nosek T.M. (2004). Defective maintenance of intracellular Ca^2+^ homeostasis is linked to increased muscle fatigability in the MG29 null mice. Cell Res..

[171] Weisleder N., Brotto M., Komazaki S., Pan Z., Zhao X., Nosek T., Parness J., Takeshima H., Ma J. (2006). Muscle aging is associated with compromised Ca^2+^ spark signaling and segregated intracellular Ca^2+^ release. J. Cell Biol..

[172] Pouvreau S., Berthier C., Blaineau S., Amsellem J., Coronado R., Strube C. (2004). Membrane cholesterol modulates dihydropyridine receptor function in mice fetal skeletal muscle cells. J. Physiol..

[173] Jiao H., Arner P., Gerdhem P., Strawbridge R.J., Näslund E., Thorell A., Hamsten A., Kere J., Dahlman I. (2015). Exome sequencing followed by genotyping suggests SYPL2 as a susceptibility gene for morbid obesity. Eur. J. Hum. Genet..

[174] Rudolf R., Khan M.M., Labeit S., Deschenes M.R. (2014). Degeneration of neuromuscular junction in age and dystrophy. Front. Aging Neurosci..

[175] Tintignac L.A., Brenner H.R., Rüegg M.A. (2015). Mechanisms regulating neuromuscular junction development and function and causes of muscle wasting. Physiol. Rev..

[176] Willadt S., Nash M., Slater C.R. (2016). Age-related fragmentation of the motor endplate is not associated with impaired neuromuscular transmission in the mouse diaphragm. Sci. Rep..

[177] Kravtsova V.V., Matchkov V.V., Bouzinova E.V., Vasiliev A.N., Razgovorova I.A., Heiny J.A., Krivoi I.I. (2015). Isoform-specific Na,K-ATPase alterations precede disuse-induced atrophy of rat soleus muscle. BioMed Res. Int..

[178] Nikolova-Karakashian M.N., Reid M.B. (2011). Sphingolipid metabolism, oxidant signaling, and contractile function of skeletal muscle. Antioxid. Redox Signal..

[179] Bryndina I.G., Shalagina M.N., Sekunov A.V., Zefirov A.L., Petrov A.M. (2018). Clomipramine counteracts lipid raft disturbance due to short-term muscle disuse. Neurosci. Lett..

[180] Yu C., Alterman M., Dobrowsky R.T. (2005). Ceramide displaces cholesterol from lipid rafts and decreases the association of the cholesterol binding protein caveolin-1. J. Lipid Res..

[181] Hardie D.G., Schaffer B.E., Brunet A. (2016). AMPK: An energy-sensing pathway with multiple inputs and outputs. Trends Cell Biol..

[182] Habegger K.M., Hoffman N.J., Ridenour C.M., Brozinick J.T., Elmendorf J.S. (2012). AMPK enhances insulin-stimulated GLUT4 regulation via lowering membrane cholesterol. Endocrinology.

[183] Carnio S., LoVerso F., Baraibar M.A., Longa E., Khan M.M., Maffei M., Reischl M., Canepari M., Loefler S., Kern H. (2014). Autophagy impairment in muscle induces neuromuscular junction degeneration and precocious aging. Cell Rep..

[184] Cerveró C., Montull N., Tarabal O., Piedrafita L., Esquerda J.E., Calderó J. (2016). Chronic treatment with the AMPK agonist AICAR prevents skeletal muscle pathology but fails to improve clinical outcome in a mouse model of severe spinal muscular atrophy. Neurotherapeutics.

[185] Khan M.M., Strack S., Wild F., Hanashima A., Gasch A., Brohm K., Reischl M., Carnio S., Labeit D., Sandri M. (2014). Role of autophagy, SQSTM1, SH3GLB1, and TRIM63 in the turnover of nicotinic acetylcholine receptors. Autophagy.

[186] Chibalin A.V., Benziane B., Zakyrjanova G.F., Kravtsova V.V., Krivoi I.I. (2018). Early endplate remodeling and skeletal muscle signaling events following rat hindlimb suspension. J. Cell. Physiol..

[187] Vilchinskaya N.A., Mochalova E.P., Nemirovskaya T.L., Mirzoev T.M., Turtikova O.V., Shenkman B.S. (2017). Rapid decline in MyHC I(β) mRNA expression in rat soleus during hindlimb unloading is associated with AMPK dephosphorylation. J. Physiol..

[188] Vilchinskaya N.A., Krivoi I.I., Shenkman B.S. (2018). AMP-Activated Protein Kinase as a Key Trigger for the Disuse-Induced Skeletal Muscle Remodeling. Int. J. Mol. Sci..

